# Self-Powered Triboelectric Ethanol Sensor Based on CuO-Doped Electrospun PVDF Fiber with Enhanced Sensing Performance

**DOI:** 10.3390/polym17101400

**Published:** 2025-05-20

**Authors:** Quanyu He, Hyunwoo Cho, Inkyum Kim, Jonghwan Lee, Daewon Kim

**Affiliations:** 1 Department of Semiconductor Engineering, Kyung Hee University, 1732 Deogyeong-daero, Giheung-gu, Yongin 17104, Republic of Korea; 2 Department of Electronics and Information Convergence Engineering, Kyung Hee University, 1732 Deogyeong-daero, Giheung-gu, Yongin 17104, Republic of Korea; 3 Department of Electronic Engineering, Kyung Hee University, 1732 Deogyeong-daero, Giheung-gu, Yongin 17104, Republic of Korea

**Keywords:** triboelectric nanogenerator, ethanol sensor, electrospinning, CuO nanoparticles, self-powered sensor

## Abstract

Electrospinning techniques have been widely applied in diverse applications, such as biocompatible membranes, energy storage systems, and triboelectric nanogenerators (TENGs), with the capability to incorporate other functional materials to achieve specific purposes. Recently, gas sensors incorporating doped semiconducting materials fabricated by electrospinning have been extensively investigated. TENGs, functioning as self-powered energy sources, have been utilized to drive gas sensors without external power supplies. Herein, a self-powered triboelectric ethanol sensor (TEES) is fabricated by integrating a TENG and an ethanol gas sensor into a single device. The proposed TEES exhibits a significantly improved response time and lower detection limit compared to published integrated triboelectric sensors. The device achieves an open-circuit voltage of 51.24 V at 800 rpm and a maximum short-circuit current of 7.94 μA at 800 rpm. Owing to the non-contact freestanding operating mode, the TEES shows no significant degradation after 240,000 operational cycles. Compared with previous studies that integrated TENGs and ethanol sensors, the proposed TEES demonstrated a marked improvement in sensing performance, with a faster response time (6 s at 1000 ppm) and a lower limit of detection (10 ppm). Furthermore, ethanol detection is enabled by modulating the gate terminal of an IRF840 metal-oxide semiconductor field-effect transistor (MOSFET), which controls the illumination of a light-emitting diode (LED). The LED is extinguished when the electrical output decreases below the setting value, allowing for the discrimination of intoxicated states. These results suggest that the TEES provides a promising platform for self-powered, high-performance ethanol sensing.

## 1. Introduction

Electrospinning has been widely adopted as a versatile fabrication technique, utilizing high-voltage electrostatic fields ranging from 5 kV to 30 kV to produce continuous fibers with diameters spanning from 50 nm to 5 μm. This method involves three critical parameters: the flow rate of the polymer solution, the applied voltage, and the working distance between the needle and collector [1,2,3]. The polymer solution is affected by the high-voltage field, and then the solution forms a Taylor cone at the tip of the electrospinning needle. Meanwhile, the jet is accelerated by the electric field and undergoes significant stretching and thinning during the process, while the solvent rapidly evaporates, and the polymer solidifies. The polymer fiber can be collected on the collector, such as a rotating drum or a stable flat stage [3,4,5]. Notably, this technology has received a lot of attention from many researchers due to its advantage in fabricating micron–submicron fibers [6,7,8]. It has a unique capability of exhibiting a high surface area-to-volume ratio and tunable porosity by adjusting the working parameters, such as temperature, humidity, and the viscosity of the polymer solution [1,9]. Recent studies have demonstrated the huge potential of electrospun fibrous films, such as biocompatible membranes [10,11,12], energy storage devices [13,14,15], and triboelectric nanogenerators (TENG) [16,17,18]. The adaptability of electrospinning technology has been shown by changing different polymers, such as using polyvinyl alcohol (PVA) [19,20,21], poly(methyl methacrylate) (PMMA) [11,17,22,23], and poly(vinylidene fluoride) (PVDF) [16,24,25]. Furthermore, some particles can be incorporated into the electrospinning process to improve the performance of the fiber for different characterizations [26] or to increase the functional capabilities of the fiber [9,27,28,29].

TENG is a device that converts different types of mechanical energy into electrical energy based on contact electrification and electrostatic induction [30]. The working method of TENG depends on the structure of the device, which has multiple shapes and various application scenarios. The components of TENG can be simply separated into electrode and dielectric layers, and both layers can be fabricated by electrospinning [7,25,31,32]. Fabrication using electrospinning with specific materials renders the TENG flexible enough to be attached to human skin, and its high biocompatibility allows TENGs to be implanted in the human body [33,34,35]. Electrospinning PVDF (ES-PVDF) fiber has been demonstrated as a flexible TENG to harvest mechanical energy from the human body and convert it into electrical energy, using the harvested electrical energy to detect the pressure and deformation of the TENG device. Due to its capability of generating electrical power, TENGs have been researched as self-powered sensors in diverse studies [36,37,38,39]. Furthermore, various sensors have been fabricated such as pressure sensors [8,40,41], stretching sensors [39,42], and gas sensors [43,44], which all show high sensitivity and reliability. The function of TENG using electrospun fiber can be modified by doping with other functional materials to increase the electrical output of TENG [31,45] or to enhance the sensing ability of the fiber [7,46].

In the fabrication of gas sensors, critical parameters including sensitivity, response time, and limit of detection must be comprehensively addressed. Copper oxide (CuO) is known by researchers for its ethanol-sensing performance. Compared with other ethanol-sensing materials like Tin oxide (SnO_2_) [47] and Zinc oxide (ZnO) [48], CuO has a lower working temperature (<100 °C) and higher scalability. Numerous studies have demonstrated that CuO exhibits stable sensing performance at room temperature and good selectivity, primarily due to its abundant oxygen vacancies, which enhance its reactive activity [49,50]. While humidity and temperature still need to be considered, as they have a significant impact on sensing performance, lower humidity can effectively reduce charge dissipation, and higher temperatures can help decrease ambient humidity, thereby enhancing sensitivity [51,52]. While CuO is known to respond to various gases such as ethanol, methanol, and certain aldehydes [53], ethanol remains the predominant component in human breath under intoxicated conditions, typically in the range of 1 ppm to 1000 ppm. Acetaldehyde is the next most abundant component, with reported concentrations ranging from 0.1 ppm to 10 ppm. Numerous studies have proposed that CuO has outstanding selectivity between these common breath compounds [54,55,56]. Based on this, and considering the relatively low concentrations of other potential interferents such as CO₂ and methane in exhaled breath, we expect their influence on the ethanol-sensing performance to be minimal. Previous investigations have highlighted the advantages of electrospun polymer fibers, particularly their remarkable surface-area-to-volume ratio and outstanding environmental stability [3,57]. While numerous studies have reported ethanol-sensing systems powered by TENGs, these implementations primarily utilized TENGs solely as power supply components [58,59,60]. To further integrate and reduce the size of the device, integrating the TENG and ethanol sensor is a feasible approach. Recent studies demonstrate the feasibility of integrating gas sensing functionality with TENG into a single device, achieving enhanced system integration. This innovative approach has yielded an ethanol response time of 240 s [61] and a limit of detection with 500 ppm [62], respectively. These results have shown a huge potential for improving sensing performance. These research findings substantiate the significant potential of TENG-integrated ethanol sensors for performance optimization through multifunctional device architecture.

In this study, we propose a self-powered triboelectric ethanol sensor (TEES) with enhanced sensing performance. Owing to nanoparticle-embedded electrospun layers with ethanol-sensing functionality, the TEES achieved enhanced sensing performance compared with previous studies. The primary dielectric layer was fabricated by an electrospinning process using PVDF fiber embedded with CuO nanoparticles, which demonstrated high sensitivity to ethanol [37,63]. Additionally, the polytetrafluoroethylene (PTFE) layer was placed under the CuO@ES-PVDF layer to enhance the electrical output of the TEES, improving both sensitivity and ethanol concentration quantification accuracy. Furthermore, this addition enhanced the electrical output, allowing the influence of noise on sensitivity measurements to be negligible at low output levels. The printed rotatable fan was positioned at the top of the dielectric layers to generate electrical energy. Various electrical output properties of the TEES are explored in detail. The primary sensing mechanism is attributed to the variation in surface charge density of the ES-PVDF layer, induced by the adsorption of oxygen molecules on the surface of ES-PVDF fibers, which is influenced by the presence of CuO particles. Furthermore, a sensitivity curve was derived for ethanol concentrations ranging from 10 ppm to 1000 ppm, covering the typical concentration range associated with human intoxication. The TEES offers advantages over commercial ethanol sensors (Portable Alcohol Tester A20, Shenzhen Aceliton Electronic Co., Ltd., Shenzhen, China) and commercial fuel-cell ethanol sensors (BACtrack S80, BACtrack, San Francisco, CA, USA), which exhibit a limit of detection of 200 ppm and 50 ppm, respectively. The TEES does not require preheating and demonstrates a faster response time. Furthermore, it operates without the need for an external power source and achieves a limit of detection comparable to that of commercial ethanol sensors. Compared to previous studies integrating TENGs with ethanol sensors, the TEES demonstrates superior performance, achieving a significantly reduced response time of 6 s and a low limit of detection of 10 ppm. A light-emitting diode (LED) installed on the device was switched off by the reduction of electrical output to indicate a higher ethanol concentration than the set value. When the exhalation of an intoxicated human is injected into the TEES, the resulting change in electrical output can be calculated and converted to sensitivity. The TEES presents a viable strategy for the integration of TENG and ethanol sensors, offering significant potential for multifunctional energy harvesting devices and advanced gas detection systems.

## 2. Materials and Methods

To fabricate the ES-PVDF mixed with CuO nanoparticles, PVDF (average Mw ~180,000 by GPC) and CuO nanoparticles (<50 nm particle size) were commercially purchased from Sigma-Aldrich (St. Louis, MO, USA). The fan, spacer, and cover of TEES were printed by a three-dimensional (3D) printer, (Bambu Lab P1S, Bambu Lab, Shenzhen, China). The bottom electrode was fabricated using printed circuit board (PCB) technology (Sample PCB, Gwangmyeong, Republic of Korea), and the design for PCB manufacturing was performed using the Altium Designer SE program (Altium, Sydney, Australia).

The parameters in the electrospinning process were important factors that affected the morphology of the fiber, such as applied voltage, flow rate, and solvent ratio of the polymer solution. The polymer solution was prepared using dimethylformamide (DMF) (Sigma-Aldrich, St. Louis, Burlington, MO, USA):acetone (SAMCHUN PURE CHEMICAL CO., LTD., Pyeongtaek, Republic of Korea) at a 1:1 ratio to dissolve PVDF beads by magnetic stirring. The CuO nanoparticles were mixed with the polymer solution after the PVDF beads were completely dissolved and continued stirring for over 2 h to be completely dispersed. Subsequently, the polymer solution was transferred into a syringe and installed on the motor in the electrospinning system (MTDI, Daejeon, Republic of Korea). The parameters of electrospinning were optimized in several experiments to fabricate uniform fibers and reduce the clumping of the CuO nanoparticles. In addition, the applied voltage was fixed at 12 kV, the flow rate was fixed at 0.54 mL/h, and the working distance was fixed at 10 cm to achieve a uniform diameter of the polymer fiber. The drum collector was used to collect the electrospun fibers and was spun at 500 rpm.

To explore the characterization of ES-PVDF fiber mixed with CuO nanoparticles, several techniques were employed to characterize the morphology and formation of the electrospinning fiber. A high-resolution field emission scanning electron microscope (Gemini 360, Carl Zeiss, Jena, Germany) and X-ray diffractometer (D8 Advance, Bruker, Billerica, MA, USA) were used to analyze the intensity and distribution of CuO nanoparticles and β-phase PVDF, respectively. An X-ray photoelectron spectroscopy (XPS) (K-Alpha plus, Thermo Fisher Scientific, Waltham, MA, USA) was used to observe the adsorbed oxygen ion on the surface of CuO@ES-PVDF. To analyze the adsorbed oxygen ions, a Fourier transform infrared spectrometer (FT-IR) (ALPHA II, Bruker, Billerica, MA, USA) was used to determine the confirmation of fabricated materials related to the surface functional groups. A bending machine was used to rotate the device at various rotation speeds. The electrical output was measured by an electrometer (Keithley Model 6514, Cleveland, OH, USA) and the Arduino Nano 33 BLE (Arduino, Ivrea, Italy) was used in tandem to transfer the electrical output value in the oxygen-less environment, which was conducted inside the glove box.

## 3. Results and Discussion

**Figure 1:**
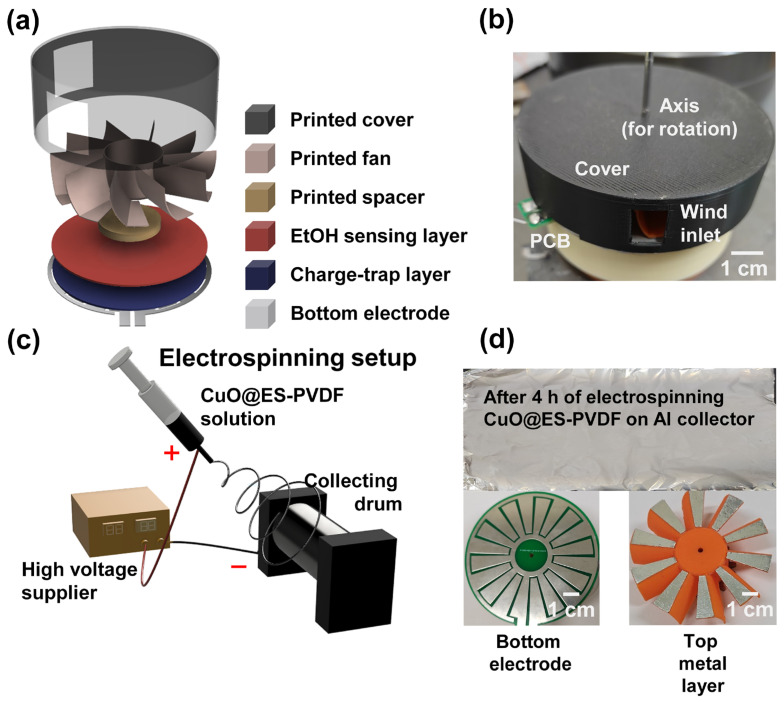
Design concepts of triboelectric ethanol sensor (TEES): (**a**) schematic image for showing the device structure of TEES; (**b**) digital camera image of TEES; (**c**) schematic diagram of electrospinning process for fabricating CuO@ES-PVDF layer; (**d**) digital camera images of CuO@ES-PVDF layer, bottom electrode, and top metal layer.

The schematic diagram of the TEES is shown in Figure 1a. The electrode of the TEES was placed at the bottom of the whole device. PTFE tape was fixed on the bottom electrode as a charge-trapping layer to increase the electrical output of the TEES. A CuO@ES-PVDF layer was fixed on the PTFE layer as an ethanol-sensing layer. The printed spacer, fan, and cover were placed successively on the CuO@ES-PVDF layer. Notably, aluminum tapes were fixed at the bottom of the fan blades of the printed fan. A steel axis was placed in the middle of the device to support the rotation of the TEES. A digital camera photo is shown in Figure 1b. The wind inlet can be seen on the side of the printed cover. The fabrication of the CuO@ES-PVDF layer via electrospinning is schematically shown in Figure 1c. The polymer solution was loaded into a syringe, and a high-voltage electric field was applied between the needle tip and the rotating drum collector. Under the influence of the electric field, polymer jets were drawn toward the collector, forming a fibrous layer. Figure 1d displays photographic images of the fabricated CuO@ES-PVDF layer, the bottom electrode, and the aluminum-coated top metal layer.

**Figure 2:**
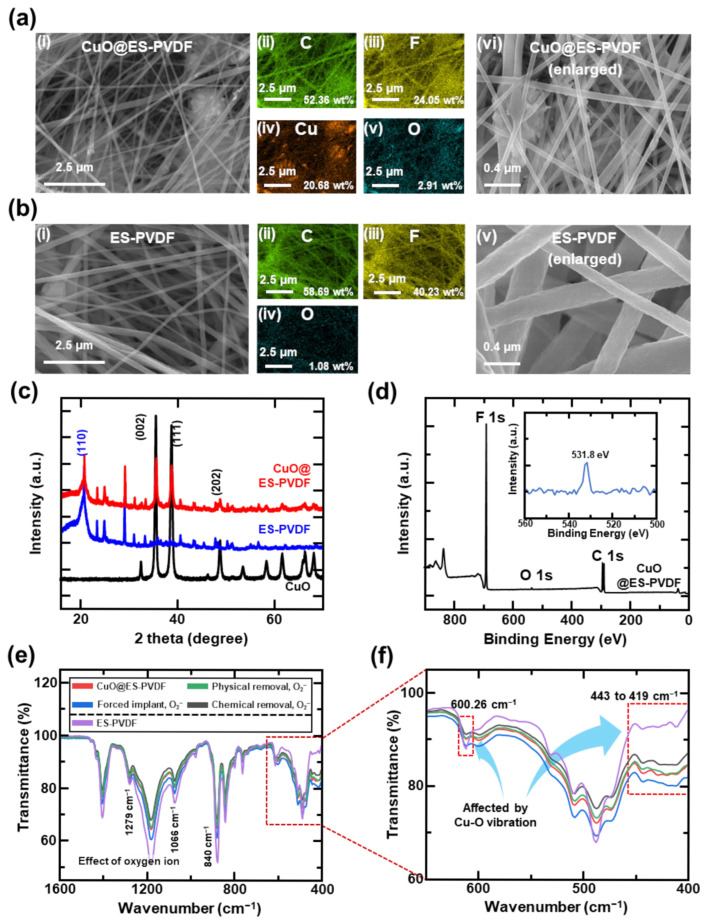
Surface characterization of CuO@ES-PVDF: (**a**) SEM images of CuO@ES-PVDF: (**i**) SEM image of CuO@ES-PVDF at large scale, (**ii**–**v**) EDS mapping result of CuO@ES-PVDF, and (**vi**) enlarged SEM image of CuO@ES-PVDF; (**b**) SEM images of ES-PVDF: (**i**) SEM image of ES-PVDF at large scale, (**ii**–**iv**) EDS mapping results of ES-PVDF, and (**v**) enlarged SEM image of ES-PVDF; (**c**) XRD patterns of CuO@ES-PVDF, ES-PVDF, and CuO; (**d**) XPS spectra of CuO@ES-PVDF; (**e**) FT-IR spectra for CuO@ES-PVDF, physically removed oxygen, chemically removed, forced implant oxygen ion of CuO@ES-PVDF, and ES-PVDF. (**f**) Enlarged spectra of FT-IR data from 400 cm^−1^ to 640 cm^−1^.

In the SEM images of Figure 2a(i), most of the CuO particles were covered by PVDF fiber. The diameter of the fiber was distributed at the submicron scale, ranging from 104.7 nm to 570.8 nm. Through energy-dispersive X-ray spectroscopy (EDS) analysis, as shown in Figure 2a(ii–v), carbon (52.36 wt%) and fluorine (24.05 wt%) were uniformly distributed within the 3D PVDF structure. Copper (20.68 wt%) and oxygen (2.91 wt%) were homogeneously dispersed in the PVDF matrix, indicating that the CuO nanoparticles were incorporated into the PVDF fiber. This architecture significantly enhanced the surface area, thereby increasing the adsorption points for oxygen molecules on ES-PVDF fibers to facilitate the formation of oxygen ions. CuO nanoparticles were uniformly distributed in the ES-PVDF fibers, as shown in Figure 2a(vi), demonstrating excellent compatibility between the PVDF solution and CuO dispersion during electrospinning. Through the EDS results, the widely distributed CuO nanoparticles with minimal clumping improved the surface area for adsorbing oxygen molecules, thereby enhancing the observation of oxygen molecules and the subsequent formation of oxygen ions. In Figure 2b(i–v), a comparison of the SEM image and EDS analysis of ES-PVDF fibers revealed no observable particles on the fiber, and the fiber appeared smoother. The SEM image further showed that the ES-PVDF fibers formed a continuous 3D network with well-defined diameters.

The XRD results of CuO@ES-PVDF, ES-PVDF, and CuO nanoparticles have been measured, as shown in Figure 2c. For the ES-PVDF fiber, a major diffraction peak occurred at a distinct angle (2θ) of 20.6°, corresponding to the (110) plane (JCPDS No. 00-038-1638), and correlated with the FT-IR spectra, as shown in Figure 2d, which typically exhibit characteristic absorption bands near 840 cm^−1^ and 1279 cm^−1^, proving that the β-phase PVDF has been fabricated successfully. Furthermore, the CuO nanoparticles exhibit distinct diffraction peaks at 2θ = 25.5°, 38.7°, and 48.7°, corresponding to the (002), (111), and (202) crystalline planes (JCPDS No. 01-072-0629). The sharp and well-defined peaks showed the high crystallinity of CuO, which is crucial for its catalytic and surface reactivity, rendering the CuO nanoparticles able to adsorb oxygen molecules and form oxygen ions more easily. The ES-PVDF fibrous layer was particularly advantageous for triboelectric applications due to its high surface area and molecular arrangement. Doping the CuO nanoparticles increased the roughness of the electrospun fiber, leading to an increase in the electrical output in the application of TENG. As shown in Figure 2d, the XPS spectra of CuO@ES-PVDF exhibit well-defined carbon and fluorine peaks, which are the primary elements of PVDF. A small-intensity peak was observed at 530.1 eV, corresponding to the O 1s signal, indicating the presence of adsorbed oxygen ions on the surface of CuO@ES-PVDF. Combined with the results of XRD analysis and SEM images, it can be inferred that the CuO particles were encapsulated by the ES-PVDF fibers. Owing to the shallow detection depth of XPS (approximately 5–10 nm), the CuO particles embedded within the ES-PVDF fibers could not be directly detected. Nonetheless, the adsorbed oxygen ions on the surface were identifiable.

In the reference paper introducing the p-type semiconductor materials [60,61,62], such as CuO [16,63,64], WO_3_ [65,66,67], and NiO [68,69,70] could adsorb oxygen molecules and form oxygen ions on their surfaces because of their oxygen vacancies [71,72,73]. Oxygen molecules adsorb electrons from the conduction band of p-type semiconductor materials. Hence, a hole accumulation layer (HAL) forms at the same time. Since the electrons are accumulated at the surface of the material, the surface conductivity of the material increases. Meanwhile, the material is prepared for sensing the target gas. When the material is exposed to the target gas, the adsorbed oxygen ions react with ethanol and water and CO_2_ is generated. With the reduction of oxygen ions, electrons return to the conduction band, and the HAL disappears at the same time. The conductivity can be reduced to the initial state. The variance in conductivity is the key data of semiconductor sensing materials for target gas sensing.

In our research, CuO particles covered by ES-PVDF fiber were adopted in this manuscript. It needed to be proven that the CuO particles could also adsorb oxygen molecules to form oxygen ions on the surface of the electrospun fiber. Therefore, the experiment was designed as shown below. The CuO@ES-PVDF fibrous film was placed in the atmosphere, and the surface adsorbed oxygen ions for over 1 h as the bare sample. The second sample was placed in a vacuum chamber and maintained in the vacuum state for over 30 min to physically remove the oxygen ions. To compare the oxygen ion density of the film, the third sample was prepared by being exposed to ethanol vapor for 1 min to remove the adsorbed oxygen ions. This process was targeted to chemically remove the oxygen ions. Finally, the last sample was measured after the O_2_ plasma treatment, during which oxygen ions were forcibly adsorbed to be compared with other samples.

Among the FT-IR data shown in Figure 2e, the bare sample exhibited a lower overall transmittance level compared to both the physically and chemically O_2_^−^-removed samples. The transmittance value increased when the oxygen ions were removed from the surface. The result proved that oxygen ions affected the transmittance data in FT-IR analysis by comparing the bare sample and the sample after O_2_ plasma treatment. The transmittance data were far lower than that of the bare sample, showing that more oxygen ions adsorbed on the CuO@ES-PVDF surface led to an increase in the infrared shift rate in FT-IR analysis [73,74]. In Figure 2f, CuO nanoparticles significantly affected the FT-IR peaks from 400 cm^−1^ to 600 cm^−1^, which is the main FT-IR peak range for CuO nanoparticles [75]. The peak differences observed in these two ranges clearly proved that CuO nanoparticles were present within the ES-PVDF. Overall, the FT-IR analysis proved that the oxygen ions can be formed on the surface of CuO@ES-PVDF and affect the transmittance level.

**Figure 3:**
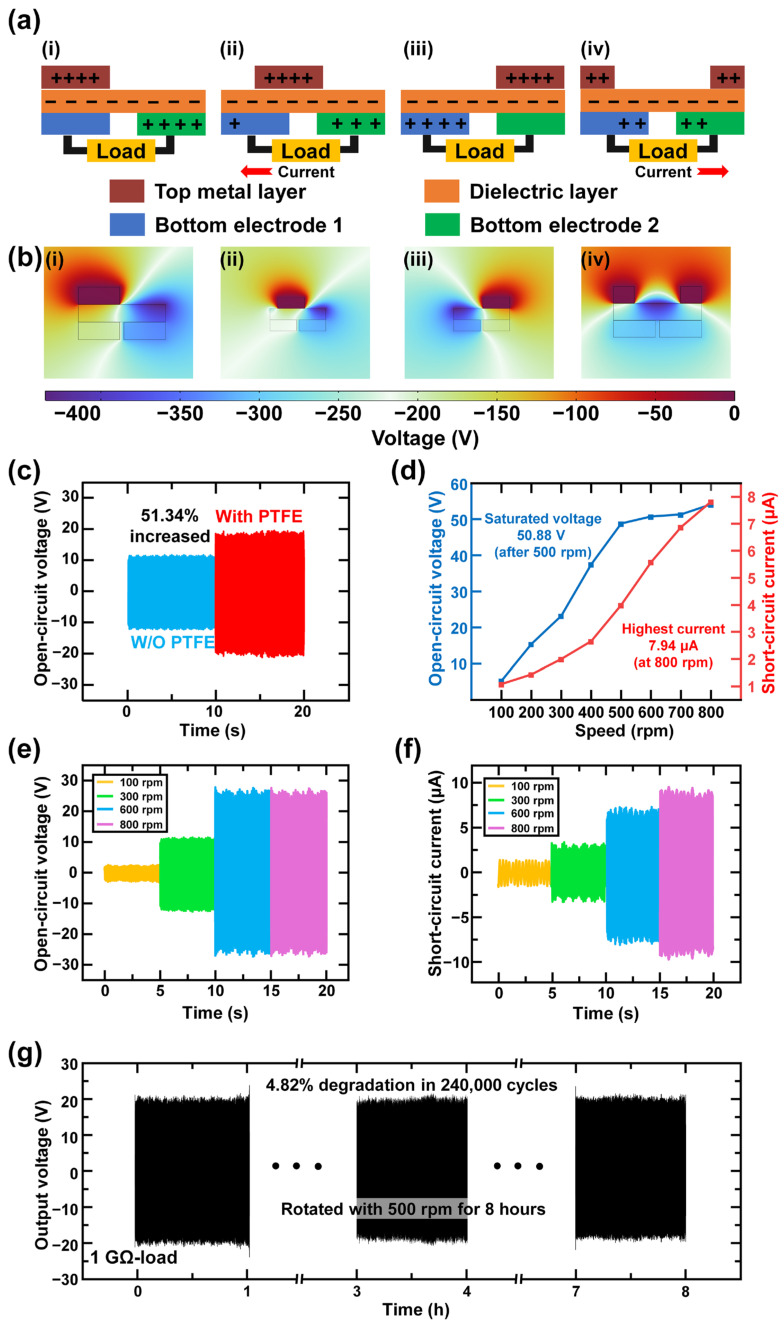
Electrical output generating mechanism and characterization of TEES. (**a**) Working mechanism of TEES: (**i**) The top metal layer (TM layer) aligns with bottom electrode 1 (BE1); (**ii**) the TM layer moves between BE1 and bottom electrode 2 (BE2); (**iii**) the TM layer aligns with BE2; (**iv**) the TM layer moves from BE2 to BE1. (**b**) Finite element method simulation of TEES: (**i**) simulation result of the TM layer aligned with BE1; (**ii**) simulation result of the TM layer moving from BE1 to BE2; (**iii**) simulation result of the TM layer aligned with BE2; (**iv**) simulation result of the TM layer moving from BE2 to BE1. (**c**) Open-circuit voltage difference with/without PTFE layer. (**d**) Electrical output trends with different input rotational speed. Output waveforms of (**e**) open-circuit voltage and (**f**) short-circuit current by varying rotational speed. (**g**) Durability result of TEES rotating with 500 rpm for 8 h.

As a freestanding TENG, TEES operated based on four stages, illustrating the top metal layer (TM layer) rotating above two bottom electrodes, as shown in Figure 3a. The PTFE layer and PVDF layer became negatively charged, while the electrodes acquired a positive charge according to the triboelectric series. When the TM layer aligned with bottom electrode 1 (BE1), as illustrated in Figure 3a(i), the charges at each layer remained in their initial state, with no charge movement between the two bottom electrodes. In Figure 3a(ii), the charges began to move as the TM layer rotated, disrupting the balance between the two electrodes. Positive charges flowed from bottom electrode 2 (BE2) to BE1, and an output current was observed at the load. In Figure 3a(iii), when the TM layer was completely aligned with BE2, the charges were fully transferred to BE1, proving that all charges were transferred and alternating current (AC) was generated in this rotating state. As the printed fan continued to rotate, the charge began to flow from BE1 to BE2, as shown in Figure 3a(iv). After the TM layer realigned with BE1 through rotation, the charge distribution returned to its initial state. The finite element method (FEM) analysis, based on COMSOL Multiphysics (version 6.2) simulation results, was conducted and displayed in Figure 3b(i–iv). The working mechanism was verified through the simulation results.

The schematic diagram of TEES is shown in Figure 1a. The bottom electrodes were fabricated using PCB technology, and a 10-grating configuration was selected for better electrical output [76]. According to the experimental data, the electrical output increased by 51.34%, from 22.60 V to 36.26 V, with the addition of the PTFE layer, as shown in Figure 3c. The ES-PVDF fibrous layer was cold-pressed under a pressure of 30 MPa for 30 min, which compressed the protruding fibers and smoothed the surface. This process improved the durability of TEES by preventing unnecessary contact between the printed fan and the dielectric layer. Additionally, a spacer was placed between the printed fan and the dielectric layer to ensure the durability of the device.

The relationship between rotation speed, open-circuit voltage (*V*_OC_), and short-circuit current (*I*_SC_) has been demonstrated in previous studies [77]. In Figure 3d–e, the output voltage showed nearly no increment from 500 rpm, and the output current increased gradually with increasing rpm. Figure 3e showed that the *V*_OC_ had reached a saturation point between the speed of charge generation and dissipation, leading to a stable output from 500 rpm, where the *V*_OC_ was 50.88 V, as shown in Figure 3d. For the *I*_SC_ case, *I*_SC_ reached the highest output of 7.94 μA when the TEES was rotating at 800 rpm. Notably, the observed enhancement in *I*_SC_ beyond 500 rpm originated from accelerated charge transfer within reduced unit time intervals, rather than an increase in the total transferred charge quantity, as shown in Figure 3f. To test the durability of the TEES, the TEES was rotated for over 8 h at 500 rpm, which corresponds to 240,000 cycles of the electrical output peaks, as shown in Figure 3g. The output voltage decreased from 41.31 V to 39.42 V, representing a reduction of 4.82%. There was a slight reduction in electrical output because of the vibration of the printed fan, which caused nearly invisible damage to the dielectric layer. The spacer effectively prevented direct contact between the printed fan and the CuO@ES-PVDF layer during electrical generation, with a thickness of over 0.25 mm. This physical isolation mechanism minimized mechanical wear induced by rotational motion, thereby achieving a prolonged operational lifespan of the TEES. The cold-pressing process of the electrospun film also affected the durability of the TEES during rotation. The electrospun film is known to many researchers for its high surface-area-to-volume ratio. Individual fibers on the surface of the electrospun film were easily damaged by the rotation of the printed fan when the space between the printed fan and the electrospun fiber layer was too small.

**Figure 4:**
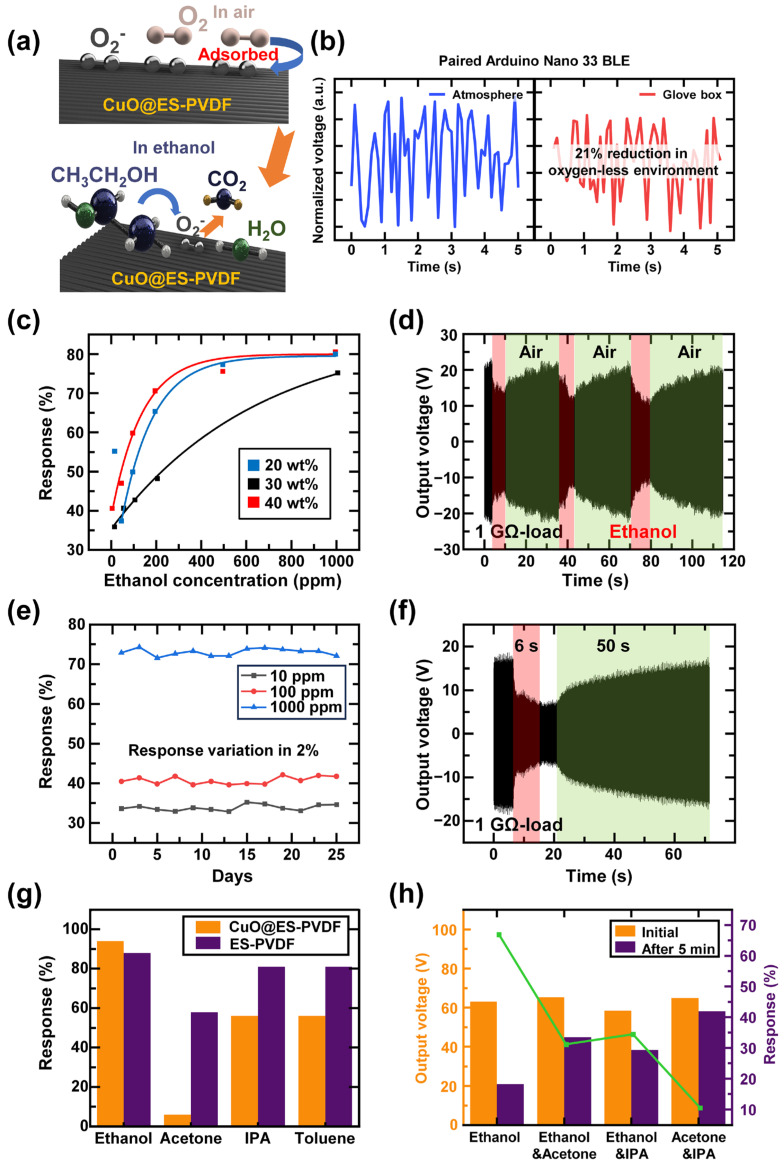
Sensing mechanism and sensing ability of TEES: (**a**) sensing mechanism of CuO@ES-PVDF in air and ethanol vapor conditions; (**b**) normalized output voltage in atmosphere and oxygen-less environment; (**c**) sensitivity curve of different CuO concentrations; (**d**) repeatability data of TEES during over 110 s; (**e**) sensing stability of TEES to 25 days of continuous test; (**f**) response time and recovery time of TEES at 1000 ppm; (**g**) selectivity data of TEES by using single volatile vapor; (**h**) selectivity data of TEES by using mixed volatile vapors.

The schematic diagram of the sensing mechanism of CuO@ES-PVDF is demonstrated in Figure 4a. Oxygen molecules were adsorbed onto the surface due to the oxygen vacancies in CuO, enabling the formation of oxygen ions. When CuO@ES-PVDF was exposed to an ethanol-containing environment, ethanol molecules reacted with the adsorbed oxygen ions, and electrons returned to the conduction band of CuO, leading to a decrease in surface charge density. The existence of oxygen ions was confirmed by FT-IR analysis, and Figure 4b illustrated the influence of CuO on the electrical output of TEES. Previous characterization experiments proved that oxygen molecules were adsorbed onto the surface of CuO@ES-PVDF and formed oxygen ions. This experiment aimed to determine the effect of oxygen ions on the electrical output. A direct current (DC) motor was used to provide a stable rotation speed for TEES, operating in both atmospheric and oxygen-depleted environments. TEES was connected to an Arduino Nano 33 BLE (Arduino Nano) and was rotated by the DC motor to generate electrical output inside a glove box with an argon-only environment. Another Arduino Nano was paired to read the output voltage from the Arduino Nano inside the glove box. As shown in Figure 4b, the output voltage exhibited a 21% reduction in the oxygen-depleted environment, which was influenced by the presence of adsorbed oxygen ions. Thus, this experiment demonstrated that oxygen ions were adsorbed by CuO nanoparticles through the ES-PVDF fiber, enabling the detection of ethanol vapor.

In the ethanol-sensing experiment, 99.99% ethanol was mixed with DI water at specific concentrations, and a humidifier was used to generate the vapor simulating the exhalation of a human in a drunk state. The TEES was exposed to various vapor conditions, and the output voltage was reduced by several seconds. The sensitivity, one of the main parameters for the sensor, was calculated by the output voltage values before and after exposure to the vapor using the following equation [50]:

(1) $$Response\;\left(\%\right)=\left(V-V_{0}\right)/V\times 100\%$$

where *V*_0_ represented the varied voltage value, and *V* was the voltage value before exposure to ethanol vapor. The electrical output of TEES decreased when exposed to ethanol-containing vapor due to the adsorbed oxygen ions reacting with ethanol molecules, generating CO_2_ and water. The surface charge density decreased because of the reduction of the HAL on CuO nanoparticles, since electrons were no longer captured by oxygen molecules and instead returned to the conduction band of the CuO nanoparticles. After the ethanol-sensing reaction, the electrical output stabilized at a specific value. Upon cessation of exposure to ethanol-containing vapor, the electrical output of TEES was restored.

To identify the appropriate sensitivity for ethanol sensing, the fiber samples with three different concentrations of CuO nanoparticles were fabricated at 20 wt%, 30 wt%, and 40 wt%. The sensitivity curves were fitted for the three different concentrations of CuO nanoparticles, as shown in Figure 4c. The results indicated that all three samples could detect the ethanol-containing vapor by the varying electrical output values. At 10 ppm, the 20 wt% composites exhibited a pronounced response of 55.38%, whereas this value significantly decreased to 38.19% at 50 ppm, defining the low limit of detection with the sensing ability. The results indicated that the 20 wt% sample could not sense the vapor below 50 ppm. For the 30 wt% and 40 wt% samples, the limit of detection improved to 10 ppm, which was better than that of the 20 wt% sample. The response at different ethanol concentrations for these two samples followed an exponential trend, and the correlation coefficients R^2^ were 0.99 and 0.98, respectively.

Considering the fabrication difficulty and recovery time, the 40 wt% sample showed higher fabrication difficulty at the electrospinning section due to its higher particle amount. CuO nanoparticles were prone to clumping together during the preparation of the polymer solution, leading to uneven distribution of CuO nanoparticles and irregular diameter of the ES-PVDF fiber. Non-uniform fibers and droplets of polymer on the electrospun film increased the recovery time of the output voltage from 30 s to more than 5 min. Therefore, the 30 wt% sample was chosen as the sensing layer of TEES.

The repeatability of TEES was investigated over a short period to verify sensing stability, as shown in Figure 4d. The output voltage was restored immediately when the ethanol-containing vapor disappeared. The sensing stability of TEES was demonstrated for 25 days at three different ethanol concentration vapors, as shown in Figure 4e. The response values varied by approximately 2% throughout the experiment, confirming the sensing stability of TEES. The chemical stability of PVDF and CuO nanoparticles ensured that the properties of CuO@ES-PVDF remained unchanged, allowing CuO@ES-PVDF to adsorb oxygen ions continuously when exposed to the atmosphere. The specific output voltage was shown in Figure 4f, the output voltage decreased immediately when the TEES contacted ethanol-containing vapor and started to recover when the TEES was no longer exposed to the ethanol-containing vapor.

To determine the selectivity for various volatile vapors, an experiment was conducted using different volatile vapors. TEES was exposed to different vapors for 5 min to evaluate its selectivity among ethanol, acetone, isopropyl alcohol (IPA), and toluene. To compare the selectivity of TEES, ES-PVDF fiber was used as a bare sample in this experiment, and the data are shown in Figure 4g. Among the experimental data, both devices exhibited significant voltage reduction when exposed to ethanol vapor, demonstrating a higher response due to the ethanol-sensing ability of TEES. The reduction in the output voltage of TEES when exposed to other vapors was lower than that of the device using the bare ES-PVDF fiber layer. The experimental data showed that ES-PVDF reduced its electrical output in various vapors. The selectivity was significantly enhanced when the ES-PVDF fiber was doped with CuO nanoparticles, and a higher response was attained when CuO nanoparticles were exposed to ethanol vapor as the sensing material. Since acetone had the highest evaporation rate among these materials, it did not significantly affect the output voltage of the TEES but showed a high response in the device using the bare ES-PVDF layer. The surface charge density decreased when vapors met the dielectric layer, as the charges were transferred to the volatile liquid molecules. This phenomenon reduced the surface charge density of TENG, leading to a decrease in electrical output. The mixed CuO nanoparticles exhibited oxygen vacancy defects, which facilitated the adsorption and dissociation of ambient oxygen molecules onto the surface of CuO@ES-PVDF, as proven in previous experimental data. This process elevated the surface charge density of the CuO@ES-PVDF layer by localizing negative charges at defect sites. The proportion of surface charges depended on the CuO nanoparticles through adsorbed oxygen ions, while volatile organic vapors that lacked reactivity with oxygen ions indirectly diminished the output of TEES by increasing ambient humidity. This phenomenon arose because non-reactive vapors primarily modulated the environmental dielectric properties, thereby accelerating charge dissipation at the surface of TEES. The results confirmed that TEES successfully identified the specific vapor in a single-vapor environment.

The subsequent experiment was conducted by mixing multiple vapors to further evaluate the selectivity of TEES, and three volatile liquids were mixed, as shown in Figure 4h. The output voltage decreased the most when using only ethanol, showing a reduction of 66.89%. When ethanol was mixed with acetone or IPA, the output voltage decreased by 31.19% and 34.76%, respectively. The output voltage exhibited the lowest reduction when a vapor mixture of acetone and IPA was used, resulting in only a 10.54% reduction in electrical output. These results proved that TEES selectively identified ethanol even when mixed with other vapors. The reduction in output voltage was primarily attributed to the interaction between ethanol molecules and CuO nanoparticles in TEES, which affected the surface charge distribution and reduced the triboelectric effect.

Table 1 provides a comprehensive comparison of the sensing performance between the proposed TEES and recent studies of self-powered ethanol sensors. Compared to previous systems using separate TENG and sensor components [25,59,60], the TEES achieved comparable sensing performance while offering integrated functionality. Owing to its lack of need to connect with a separate sensor, the TEES can achieve higher compatibility. The results demonstrated that the TEES exhibited superior performance to prior integrated TENG-ethanol sensors [61,62], achieving a significantly lower limit of detection and faster response time. Notably, the TEES maintained comparable sensitivity and response time to those parameters of separated self-powered sensors while offering the additional advantage of system integration.

**Figure 5:**
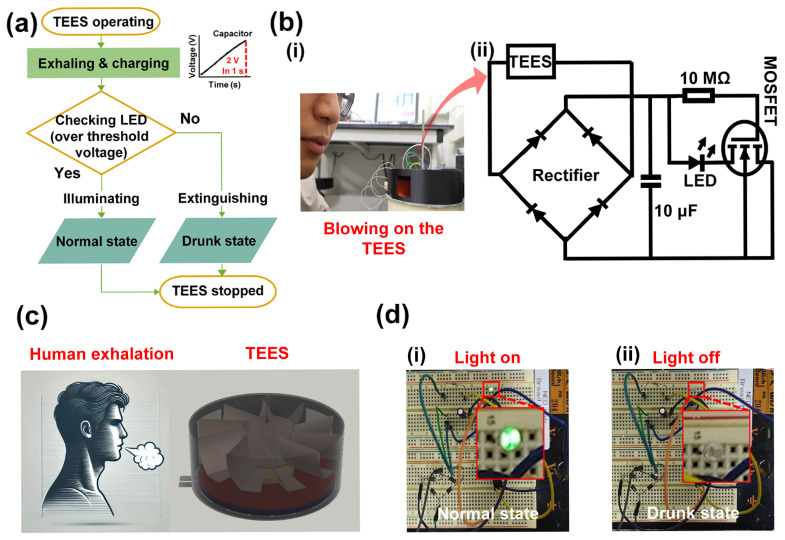
Application of TEES as a self-powered ethanol sensor: (**a**) flow chart of self-powered ethanol sensing; (**b**) (**i**) digital photograph of test and (**ii**) circuit diagram for TEES system; (**c**) schematic diagram of implementation for TEES with exhalation to detect human drunk state; (**d**) circuit set up images on the breadboard and confirmation of illuminating an LED.

To demonstrate the self-powered capability of TEES, the flow chart was placed in Figure 5a. When human exhalation blew onto the TEES to rotate the printed fan, the capacitor started to be charged. Meanwhile, the ethanol concentration could be checked by the illumination of the LED. The extinguished LED demonstrated that the ethanol concentration of the human exhalation was over the set limit. The digital photograph captured during testing is presented in Figure 5b(i), and the circuit diagram is shown in Figure 5b(ii). The TEES was connected to a full-bridge rectifier to provide a DC output. A 10 μF capacitor was connected to stabilize the electrical output. A 10 MΩ resistor was connected to the gate side of an IRF840 metal-oxide-semiconductor field-effect transistor (MOSFET) to divide the output voltage from TEES, and an LED was connected to the drain terminal of the MOSFET. The source terminal of the MOSFET was connected to the negative side of the rectifier.

As illustrated in Figure 5c, if the ethanol concentration was less than the set value, the LED was illuminated by the TEES output under normal conditions. In this state, the gate terminal of the MOSFET received sufficient voltage to maintain the drain-source connection, thereby enabling the illumination of the LED. However, upon exposure to ethanol vapor, the TEES output decreased by over 40% when the ethanol concentration exceeded 100 ppm, causing the voltage at the gate terminal to drop below the required voltage to sustain the on-state of the MOSFET. Consequently, the drain-source circuit was interrupted, and the LED was extinguished, providing a visual indication of ethanol detection. In Figure 5d(i,ii), the illumination and extinguishing of the LED were clearly observed. When a person in a drunken state blew onto the TEES, the variation in output voltage affected the illumination of the LED, indicating the person’s level of intoxication.

## 4. Conclusions

In summary, a self-powered ethanol sensor combining the TENG and ethanol sensor into one single device is proposed. The ES-PVDF fiber is mixed with CuO nanoparticles, providing the main function for ethanol sensing, and the embedding of the PTFE layer is targeted to increase the electrical output to make sensitivity calculation easier. The electrical output of TEES is explored, with the *V*_OC_ reaching 50.88 V at 500 rpm and the *I*_SC_ reaching 7.94 μA at 800 rpm. The spacer ensures the excellent durability of the TEES by avoiding contact between the printed fan and the CuO@ES-PVDF layer, resulting in only a 4.82% reduction in electrical output. Response time, sensitivity, selectivity, and stability are explored in detail, which proves the excellent sensing performance of TEES. Compared to previous studies, the TEES demonstrates a faster response (6 s at 1000 ppm) and higher sensitivity (10 ppm detection limit). The electrical output from TEES is able to illuminate the LED by using a MOSFET to accomplish automatic ethanol sensing. This research is expected to promote the development of self-powered ethanol sensors. This research demonstrates significant potential for integrated self-powered gas sensors and suggests that the volume of the TENG device can be further reduced, offering broad application prospects.

## Figures and Tables

**Table 1:** Comparison of the sensing performance of the triboelectric ethanol sensor based on CuO@ES-PVDF with previously reported devices, including both separated TENG-ethanol sensor systems and integrated single-device architectures.

Sensing Material	Device Type	Response Time	Limit of Detection	Reference
ZnO nanowire/Ag	Separated	20 s (5 ppm)	5 ppm	[25]
WO_3_ nanorods	Separated	5 s (100 ppm)	5 ppm	[59]
β-Ni(OH)_2_/MXene	Separated	15 s (100 ppm)	5 ppm	[60]
ZnO/PTFE layer	Integrated	240 s (2%)	10 ppm	[61]
Polyimide nanowire	Integrated	28 s (10,000 ppm)	500 ppm	[62]
CuO@ES-PVDF	Integrated	6 s (1000 ppm)	10 ppm	This work

## Data Availability

The original contributions presented in this study are included in the article. Further inquiries can be directed to the corresponding author.
